# Research on the anti-ageing mechanism of *Prunella vulgaris* L.

**DOI:** 10.1038/s41598-023-39609-1

**Published:** 2023-07-31

**Authors:** Ping Li, Xiao Lv, Junrong Wang, Chenyang Zhang, Jiahao Zhao, Yadong Yang

**Affiliations:** 1 School of Laboratory Medicine and Bioengineering, Hangzhou Medical College, Hangzhou, 311399 China; 2 Dian Diagnostics Group Co., Ltd, Hangzhou, China; 3 Key Laboratory of Digital Technology in Medical Diagnostics of Zhejiang Province, Zhejiang, China; 4 The Affiliated Yixing Hospital of Jiangsu University, Yixing, China; 5 Department of Clinical Laboratory, The Affiliated Yixing Hospital of Jiangsu University, Yixing, China

**Keywords:** Cell biology, Drug discovery, Molecular biology, Medical research, Molecular medicine

## Abstract

*Prunella vulgaris* L. (*P. vulgaris*) has long been considered to have antipyretic, analgesic and anti-inflammatory effects, lowering blood lipids and pressure. Many studies show that in addition to the traditional telomere attrition, DNA damage and epigenetic changes, immunosenescence is also a new possibility to explore the mechanism of ageing. Therefore, this herb may have potential anti-ageing effects. Typically, there are a series of markers that identify senescent cells, such as superoxide dismutase (SOD)2, an inhibitor of CDK4 (p16^INK4A^), tumor necrosis factor (TNF)-α, immune cells number, proliferation, and nuclear abnormalities. These changes rarely present in young tissues, while greatly increasing in response to ageing. Firstly, the ageing model of the Institute of Cancer Research (ICR) mouse was established by d-galactose subcutaneous injection. Then, SOD2, p16^INK4A^ and TNF-α were detected by quantitative Real-time PCR (qPCR), Western Blot (WB) and Enzyme-Linked Immunosorbent Assay (ELISA). Simultaneously, senescent cells in livers were stained by hematoxylin and eosin (HE). The viability of splenocytes was detected by Cell Counting Kit-8(CCK-8). The difference in specific immune cells (NK cells, B lymphocytes and T lymphocytes) was detected by flow cytometry. Both low (100 mg/kg) and high (300 mg/kg) concentrations of *P. vulgaris* treated ageing ICR mice show anti-ageing alterations, such as p16^INK4A^ decreased approximately 1/2 and SOD2 tripled in livers, TNF-α decreased from 1 to 0.6 in plasma, and T cells increased from 0.09 to 0.19%. Compared with the ageing group, the spleen cells in the Prunella-treated group had stronger proliferation ability. Thus, *P. vulgaris* could have an anti-ageing effect. This is the first study to demonstrate the anti-ageing effect of *P. vulgaris*. It may also be capable of preventing a variety of age-related diseases.

## Introduction

Ageing always accompanies chronic diseases and disabilities, which negatively affects the society and healthcare costs^1^. For instance, the prevalence of type 2 diabetes rises with age, and diabetics are prone to infections due to nerve damage, which can lead to irreversible consequences such as amputation and disability^2–4^. The cost of treating diabetes worldwide is anticipated to exceed $490 billion by 2030^5^. The common characteristics of ageing mainly include genome instability, telomere attrition, epigenetic changes, proteostasis loss, disabled macroautophagy, deregulated nutrient-sensing, mitochondrial imbalance, stem cell exhaustion, altered intercellular communication, chronic inflammation, microbial imbalance^6^. Cellular ageing is generally present in the ageing of individuals which is an irreversible state in which the cell cycle stops, if ageing cells are not cleared promptly, the accumulation of these cells can lead to inflammation and the development of tumors^7^. With the development of contemporary clinical medicine, we gradually realize that chronic inflammation is a major hallmark of ageing and can accelerate the ageing process, which can be slowed down by anti-inflammation to produce an anti-aging effect^8^. In addition, ageing usually coupled with modifications in immune-related markers: (1) At the molecular level, an inhibitor of CDK4 (p16^INK4A^), superoxide dismutase (SOD)2, and tumor necrosis factor (TNF)-α may have significantly changed as cells grew older; (2) At the cell level, the morphology and structure of senescent cells also undergo significant changes, such as enlargement of the cytoplasm and nucleus, an increase in cytoplasmic granules, folding of the nuclear membrane, condensation of chromatin, and loss of three-dimensional structure; (3) at the individual level, ageing can lead to slower wounds healing, wrinkles increasing, appetite-reducing, and slower reactions^9^.

According to Burska et al.^10^, the anti-TNF-α antibodies can considerably lower insulin resistance and improve insulin sensitivity in patients with rheumatoid arthritis (RA). Etanercept, a TNF-inhibitor, dramatically decreased the probability of Alzheimer’s disease development in RA patients^11^. Ageing is related to the activity of inflammatory factors in the blood, like TNF-α^12,13^. TNF-α is a multifunctional pro-inflammatory cytokine, and plasma concentrations of TNF-α rise with age^13^.

*Prunella vulgaris* L. (*P. vulgaris*) is a perennial herb in Prunella^14^. It is a traditional Chinese medicine widely used to treat inflammation, ophthalmodynia, headache and cancer^15,16^. The probable mechanisms behind its antiviral, antibacterial, anti-inflammatory, immunomodulatory, antioxidant, and anti-tumor effects have also been elucidated by contemporary pharmacological research^17,18^. *P. vulgaris* is the main ingredient in herbal tea. In vivo, studies have shown that drinking herbal tea can alleviate the immune function decline caused by restraint stress in mice, which is manifested by lymphocyte damage reduction and the increase of the number and activity of natural killer (NK) cells and T lymphocytes^19^. In vitro, both aqueous and ethanol extracts of *P. vulgaris* have been proven in studies to enhance lymphocyte proliferation and control cytokine production^19,20^. However, *P. vulgaris* has not been linked to any reports of an anti-aging effect previously. The study’s aim is to demonstrate the effect of *P. vulgaris*.

When Song et al. initiated the establishment of an evaluation of the d-galactose-induced ageing model via injection^21^. Numerous studies have demonstrated that long-term massive injection of d-galactose solution can produce ROS, advanced glycosylation end products (AGE), reduce the activity of antioxidant enzymes, form more superoxide anions and various oxidation products, leading to the functional decline in a variety of organs, including brain, myocardium, liver, kidney, muscle, blood vessels and ovary^22–27^. Therefore, in this study, d-galactose induction was selected to build the ageing model.

## Materials and methods

### Purification of *Prunus vulgaris*

The dried *P. vulgaris* (Minsheng Pharmaceutical Group Co., Ltd. China) was weighed, cleaned once with deionized water, and soaked with 40 °C deionized water at a solid–liquid ratio of 1:25 (g/ml) for 1 h. Then, the soaked *P. vulgaris* was heated (100 °C) 2 times (90 min), and the decoctions obtained from the two times were mixed and concentrated by heating (100 °C, 30 min). Next, the liquid from the last step is filtered via a 0.22 μm mixed cellulose ester filter membrane. Slowly add 95% ethanol solution into the crude filtered liquid while stirring, so that the final concentration of ethanol reaches more than 80%, and stand overnight at 4 °C refrigerator. After centrifugation at 1570 g for 10 min, the supernatant was heated and concentrated to fully evaporate the ethanol. The crude drug content was calculated according to the raw drug input amount. The crude drug concentration generally refers to the amount of crude medicine (g)/volume of liquid medicine (ml)], the concentration of alcohol extract concentrated solution was 3 g/ml.

### Constructing the ageing model

After 1 week of adaptive feeding, 36 ICR mice (half male and half female), approximately 8 weeks old and weighing 28–31 g, were randomly assigned to the d-galactose group or control group (saline). The ageing mouse model was constructed as follows: 4 g d-galactose powder was weighed and dissolved in 200 ml sterile distilled water and autoclaved in a pressure cooker, and then 0.5 g/kg was injected subcutaneously into the neck and back of ICR mice for 35 consecutive days. In contrast, control mice were treated with sterile saline.

### *P. vulgaris* treatment

Thirty-six mice were divided into the following groups: high-dose (300 mg/kg *P. vulgaris* and d-galactose 0.5 g/kg) and low-dose (100 mg/kg *P. vulgaris* and d-galactose (0.5 g/kg) groups, negative control group (0.5 g/kg d-galactose), positive control group (25 mg/kg Vitamin E and d-galactose (0.5 g/kg), and blank control group (normal saline, NS). The anti-ageing study was carried out continuously for 55 days.

### Quantitative real-time PCR (qPCR)

Total RNA was extracted from tissues using TRIzol according to the instruction, firstly 1 μg total RNA was reverse transcribed using the RT kit (#7E542I1, Vazyme, Chain) in the MiniAmp Thermal Cycle Thermal cycler (AB Applied Biosystems, Singapore). qRT-PCR was repeated three times using SYBR Premix Ex Taq™ II (#820A, TaKaRa, Japan) in a LightCycler 480 system (Roche, Switzerland). GAPDH was used as an internal reference gene for standardization. The following qPCR primers were synthesized by Shanghai Generay Biotech Co., Ltd. (Shanghai) (SOD2: F:5ʹTGGGAGTCCAAGGTTCAG3ʹ; R:5ʹGATTAGAGCAGGCAGCAA3ʹ; P16^INK4A^: F:5ʹCGAACTCGAGGAGAGCCATC-3ʹ; R:5ʹTACGTGAACGTTGCCCATCA3ʹ; TNF-α: F:5ʹACCCTCACACTCACAAACCA3ʹ; R:5ʹACCCTGAGCCATAATCCCCT3ʹ; GAPDH: F:5ʹTATGACTCCACTCACGGCAAAT3ʹ; R:5ʹGTCTCGCTCCTGGAAGATGG3ʹ).

### Western blotting (WB)

Total protein was extracted from the liver and kidney. Approximately 20 ng of protein from each sample was loaded onto a 15% SDS-acrylamide gel. The proteins are separated and transferred to a nylon membrane at a constant voltage. Then, the membrane was incubated with rabbit anti-SOD2 antibody, rabbit anti-P16^INK4A^ antibody (#13268-1-AP, 1:1000 dilution; Proteintech Group Inc., USA) and mouse anti-GAPDH monoclonal antibodies (#60004-1-Ig, 1:1000 dilution, Proteintech Group Inc., USA) overnight at 4 °C. The membranes were washed three times with (TBST) and incubated with luciferase-conjugated anti-rabbit, anti-mouse IgG for 2 h at room temperature (RM). Image Studio 2.0 (Li-Cor Odyssey Clx, USA) was used for quantitative analysis.

### HE staining

Liver (4 μm) from paraffin blocks were stained by hematoxylin and eosin. Views were randomly selected and photographed by the light microscope (Olympus Japan) at × 100 magnification.

### Enzyme-linked immunosorbent assay (ELISA)

Serum samples from each group were analyzed in duplicate according to the manufacturer’s instructions (Shanghai Jianglai Industrial Limited By Share Ltd), and absorbance was measured at 450 nm. Standard curves were generated by plotting absorbance values against concentration gradients to measure serum concentrations of TNF-α in each group.

### Malondialdehyde (MDA) determination

According to the manufacturer’s instructions (Beijing Solarbio Science & Technology Co., Ltd). Serum samples from each group were analyzed in duplicate. The absorbance was measured at 450 nm and 600 nm wavelengths, respectively. Standard curves were generated by plotting absorbance values against concentration gradients to measure MDA levels in the serum of each group.

### Splenocyte proliferation assay (CCK-8)

Cells from the spleen were cultured with DMEM (ThermoFisher Biochemical Products Co., Ltd), 10% fetal bovine serum (Yeasen Biotechnology Chain Co., Ltd), 1% anti-penicillin–anti-streptomycin (Shanghai Generay Biotech Co., Ltd) in a 5%CO2 cell incubator. The absorbance was measured at 450 nm. Standard curves were generated by plotting absorbance values and concentration gradients to detect the proliferation ability of splenocytes in each group.

### Flow cytometry

100 ul anticoagulant blood was incubated with four kinds of antibodies, namely BV421—anti-CD4 antibody (#562891, BD Pharmingen, USA), APC-CY7—anti-CD3 antibody (#560590, BD Pharmingen, USA), FITC-anti-CD8 antibody (#553030, BD Pharmingen, USA), PE—anti-NK1.1 antibody (#553165, BD Pharmingen, USA) and APC—anti-CD19 antibody (#550992, BD Pharmingen, USA) (RT,30 min, keep in the dark). Next, 0.5 ml red blood cell lysate (Hangzhou Simgen Biotechnology Co., Ltd) was added (RT, in the dark) and centrifuged at 1200 rpm for 5 min, and the supernatant was discarded. After mixing with 1 ml PBS (Beijing Solarbio Science & Technology Co., Ltd), the supernatant was discarded again. Finally, the cell was resuspended in 400 ml PBS. Flow cytometry was performed on a Becton Dickinson FACS instrument (BD, USA).

### Statistical analysis

GraphPad Prism 8.0.1 was used for statistical analysis. Data are expressed as the mean ± standard deviation (SD) in triplicate. The student t-test was performed to compare with the control group, and one-way ANOVA analysis with a significance level of p < 0.05 was used. Comparisons were made between different treatment groups.

### Methods of euthanasia

Cervical Dislocation as a humane euthanasia method in mice.

Each experimental group contained at least 3 mice. All experiments were approved by the Experimental Animals Committee, Hangzhou medical college, and were performed in accordance with the institutional guidelines and Guidelines for Proper Conduct of Animal Experiments by the ARRIVE guidelines.

Experiments on plants comply with institutional, national, and international guidelines.

## Results

### d-Galactose induces ageing and changes ageing-related markers expression in various organs

**Figure 1:**
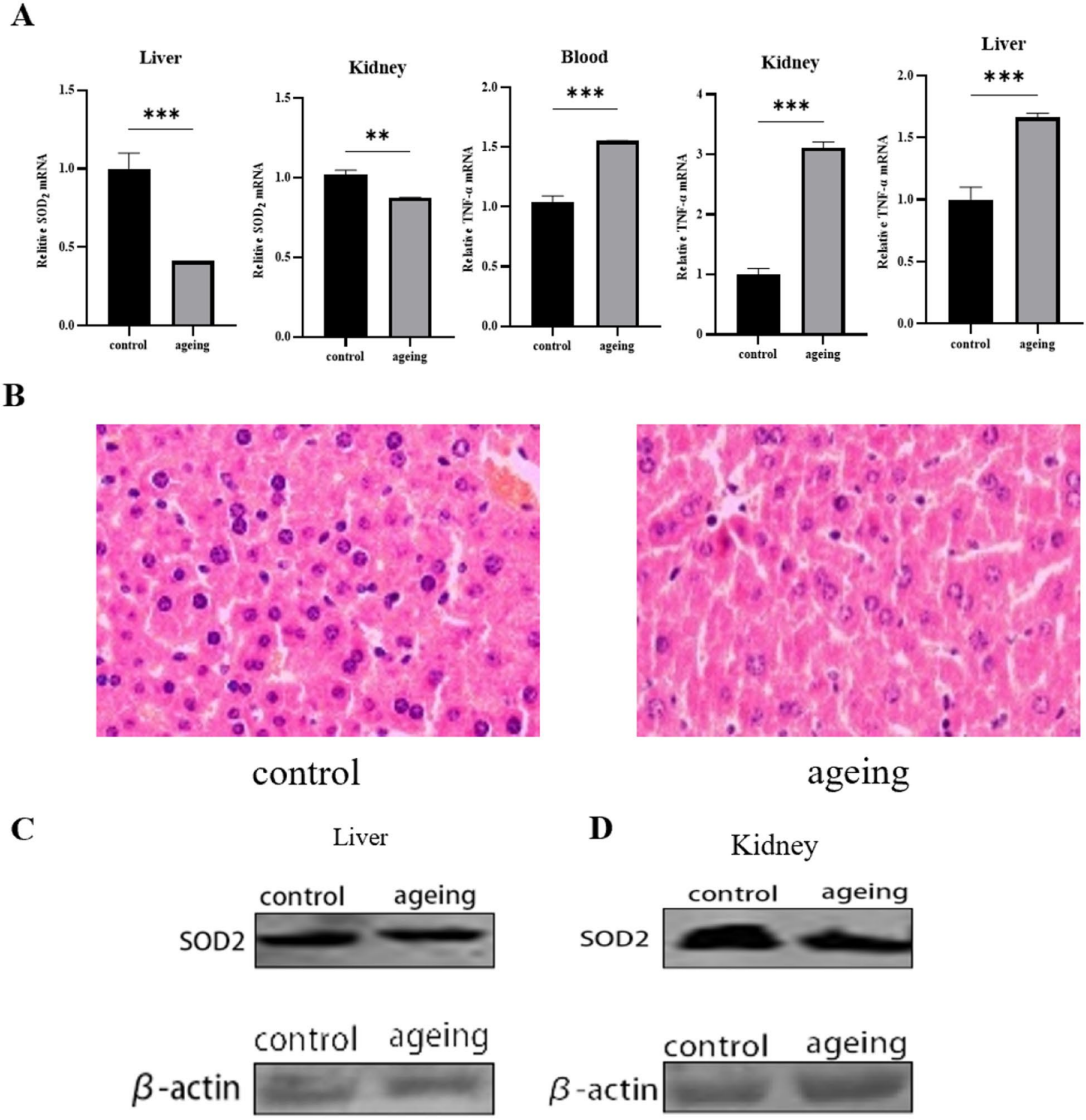
d-Galactose induces ageing and changes ageing-related markers expression in various organs. (**A**) RNA was extracted from the liver and kidney of mice to detect the expression level of the SOD2 gene, and the expression level of TNF-α in serum, liver and kidney of mice was detected; (**B**) the liver of mice was sliced and stained with HE; (**C**) the expression level of the SOD2 protein in the liver tissues of mice was detected; (**D**) the expression level of the SOD2 protein in the kidney tissues of mice was detected. *Represents p < 0.05, **represents p < 0.01 and ***represents (p < 0.001).

The mice in the experimental group were subcutaneously injected with d-Galactose (0.5 g/kg), while the mice in the control group were subcutaneously injected with 0.09% NS. After 35 days of continuous treatment, it can be clear seen that the mouse in the ageing group has thinner and coarser hair and slower reaction than the NS-treated control group. However, some ageing-related markers are differentially expressed. SOD2 in ageing mice’s liver and kidney is significantly lower than that of the NS group in transcriptional (p < 0.001) (Fig. 1A). HE staining in their livers has similar results. In the ageing mouse, the nuclei are larger, and have obvious nuclear fragmentation and dissolution (Fig. 1B). The expression of TNF-α in the liver, kidney, and plasma of the ageing group almost increased 1.5 times, 3 times and 1.5 times, respectively, when compared with the NS group (Fig. 1A). Compared to the NS group, the expression of SOD2 protein in the liver and kidney was reduced in the D-galactose group (Fig. 1C,D). Significant differences in both ageing-related markers in the two groups directly reflect that the ageing model is built successfully.

### Different doses of *P. vulgaris* not only *rejuvenated* the splenocyte function but also changed the number of immune cells

**Figure 2:**
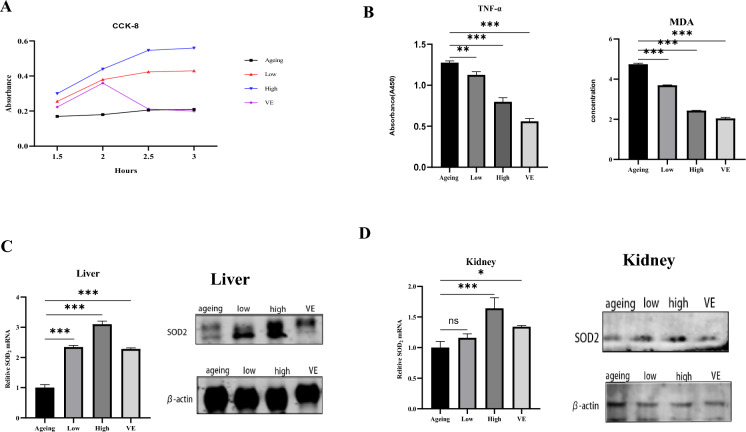
Different doses of *P. vulgaris* not only *rejuvenated* the splenocyte function but also changed the number of immune cells. (**A**) The proliferation ability of spleen cells in each group was detected; (**B**) contents of TNF-α and MDA in serum of mice in each group; (**C**) the expression levels of SOD2 at the gene level and protein level in the liver of mice in each group; (**D**) the expression levels of SOD2 in the kidney of each group were detected at the gene level and the protein level. Where *represents (p < 0.05), **represents (p < 0.01), ***represents (p < 0.001).

The spleen is a crucial immunological organ in the human body that harbors a significant population of lymphocytes involved in immune responses. When bacteria or viruses invade the body, they will be recognized by lymphocytes, engulfed, and finally removed from the spleen. Therefore, we tested the proliferation ability of mouse spleen cells in different groups. Compared with other groups, it was found that although the proliferation ability of splenocytes in mice treated with VE was decreased after 2 h of CCK8 treatment, the ability in low-dose *P. vulgaris*, high-dose *P. vulgaris* and the ageing group was increased. Interestingly, compared with the ageing group with weak proliferation ability, the proliferation ability in two *P. vulgaris* -treated groups were greatly enhanced, and the proliferation ability increased with the increase of *P. vulgaris* dose (Fig. 2A).

To further investigate the effect of *P. vulgaris* on the number of immune cells, flow cytometry analysis was performed on T (CD3^+^CD4^+^ or CD3^+^CD8^+^) lymphocytes, B (CD3^−^CD19^+^) lymphocytes and NK (CD3^−^NK1.1^+^) cells in the blood of each group. The results showed that the number of CD4^+^T lymphocytes increased from 0.35 to 0.39% and 0.38% respectively, and CD8^+^ T lymphocytes increased from 0.09 to 0.19% and 0.15% respectively after treatment with low and high concentrations of *P. vulgaris*, while in the VE group, there shows a downward trend. The reason for this may be that *P. vulgaris* is more effective than VE in reversing senescence. As for B lymphocyte, the tendency in both doses *P. vulgaris* group and the VE group are increasing. B lymphocytes in the high concentration group doubled that in ageing group. Interestingly, the results for NK cells were different. NK cells decreased in the low-concentration group, however, increase in the high-concentration group and the VE group. NK cells in the high-concentration group nearly tripled that in the ageing group, which means that high-concentration *P. vulgaris* has the best anti-ageing effect in the bloodstream (Table 1).

**Table 1:** The effect of *P. vulgaris* on the CD4^+^T lymphocyte, CD8^+^ T lymphocyte, B lymphocyte and NK cell.

	CD3+ CD4+ T lymphocyte		CD3+ CD8+ T lymphocyte		CD3−CD19+ B lymphocyte		CD3−NK1.1+ NK cell	
	Count	Total (%)	Count	Total (%)	Count	Total (%)	Count	Total (%)
Ageing	2832	0.351428	785	0.097412	1344	0.166779	41	0.005088
Low	1327	0.394517***	703	0.191887***	682	0.185609**	18	0.004913 ns
High	3363	0.389279***	1364	0.157888***	2966	0.343325***	144	0.016668***
VE	2819	0.199767***	1320	0.093541 ns	2430	0.172201 ns	74	0.005244 ns

**represents (p < 0.01), ***represents (p < 0.001).

### *P. vulgaris* treatment changes the levels of different ageing markers in different organs and leads to morphological changes

Serum levels of TNF-α and MDA were detected after 55 days of treatments. TNF-α in the serum decreased by 0.2-fold (p < 0.01) and 0.4-fold (p < 0.001) respectively after treatment with low and high concentrations of *P.vulgaris,* and the MDA decreased by onefold (p < 0.001) and twofold (p < 0.001) respectively (Fig. 2B). The levels of SOD2 in the liver increased by 2.3-fold (p < 0.001) and 3.1-fold (p < 0.001) respectively after treatment with low and high concentrations of *P. vulgaris* (Fig. 2C), and in the kidney, it increased by 1.1-fold (p > 0.05) and 1.64-fold (p < 0.001) respectively (Fig. 2D), while the anti-ageing effect of the low-dose and VE groups was equal.

### After *P. vulgaris* treatment, hepatocytes showed morphological changes of *reverse ageing*

**Figure 3:**
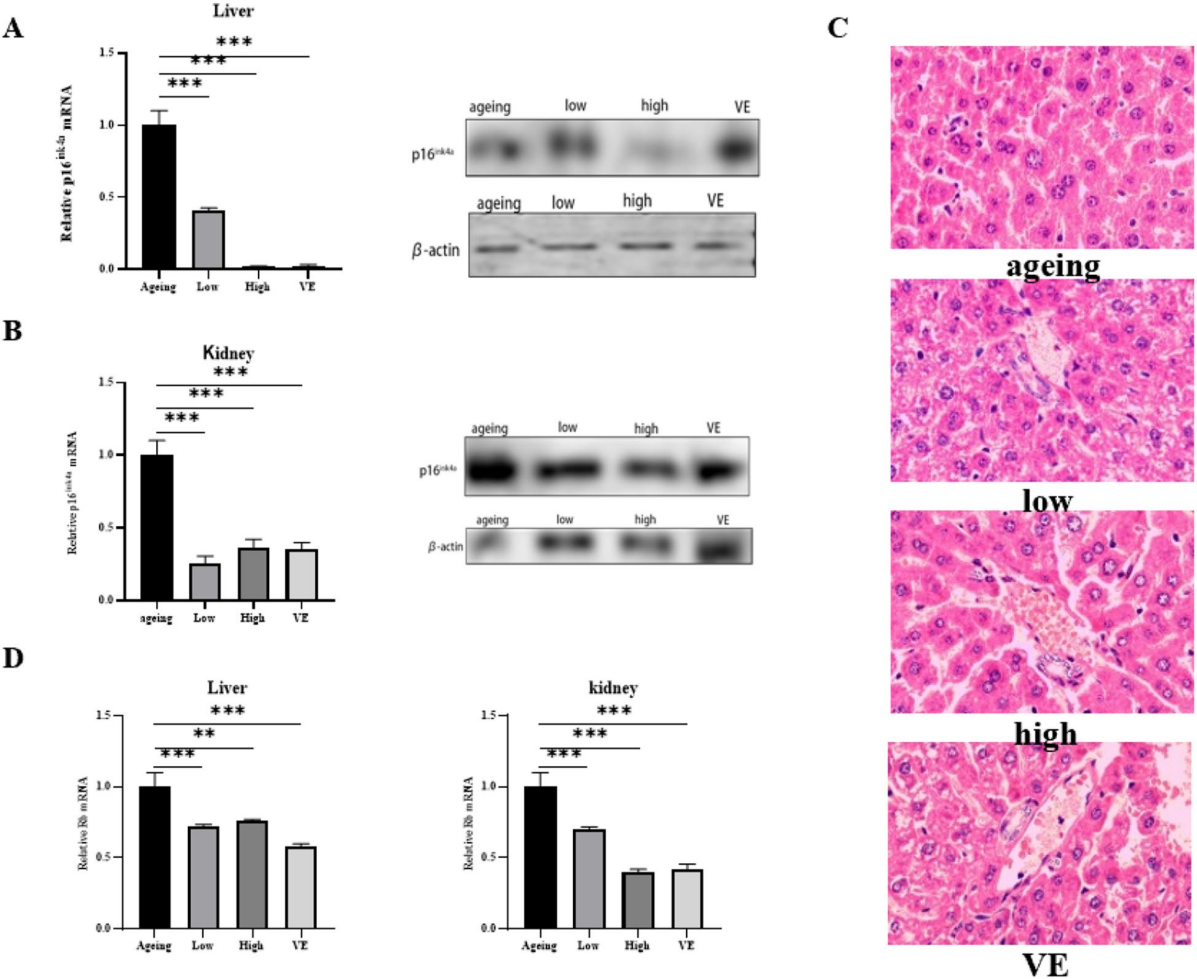
After *P. vulgaris* treatment, hepatocytes showed morphological changes of *reverse ageing* (**A**) the expression levels of p16^INK4A^ at the gene level and protein level in the liver of mice in each group; (**B**) the expression levels of p16^INK4A^ in the kidney of each group were detected at the gene level and the protein level; (**C**) liver of mice in each group were sliced and stained with HE; (**D**) the phosphorylated Rb expression in liver and kidney of mice in each group was detected. Where *represents (p < 0.05), **represents (p < 0.01), ***represents (p < 0.001).

We also detected p16^INK4A^ in different organs of each group. Compared with other groups, p16^INK4A^ in the liver decreased by 0.5-fold (p < 0.001) and 0.8-fold (p < 0.001) respectively after treatment with low and high concentrations of *P. vulgaris* (Fig. 3A), while in the kidney, it decreased by 0.7-fold (p < 0.001) and 0.6-fold (p < 0.001) respectively (Fig. 3B). Its downstream protein Rb was also detected, and its expression was reduced in both the liver and kidney (Fig. 3C).

Besides, livers in each group were dissected and stained with HE. Compared with other treatment groups, it was found that liver cells in the high-dose group were more intact, with obvious nuclear membrane, obvious liver lobe orientation, sinus hyperemia, and almost no apoptotic bodies (Fig. 3D).

## Discussion

Currently, a number of important traits are commonly acknowledged as crucial signs of ageing. The oxygen free radical hypothesis proposed by Denham Harman: the accumulation of harmful oxygen free radicals produced by body metabolism eventually cause cell ageing. Telomere hypothesis: In normal somatic cells, due to the absence of telomerase, the telomeres at the end of chromosomes will be shortened once for each replication of genetic material. The ageing of cancer cells can be caused by telomerase inhibition. The hypothesis of immune ageing proposed by Walford: The immune system’s ability to respond to antigens will be reduced by changes in the quantity and function of T lymphocytes, B lymphocytes, NK cells, dendritic cells (DC), macrophages, and neutrophils, which will increase the risk of infection, autoimmune disease, and malignant tumors. Genome stability: Accumulation of genomic damage accompanies the ageing process, while artificially induced genomic damage can trigger certain accelerated ageing manifestations. Epigenetic alterations: Ageing is accompanied by epigenetic changes, and epigenetic disruption can result in premature ageing syndromes in model organisms. Loss of proteostasis: During the ageing process, changes in protein homeostasis occur, characterized by chronic expression of unfolded or misfolded proteins, or protein aggregates. These changes can promote the development of certain age-related disorders, like Alzheimer’s disease, Parkinson’s disease, and cataracts. Deregulated nutrient sensing: Synthetic metabolic signals accelerate ageing while decreasing nutrient signals can extend lifespan. Stem cell exhaustion: stem cell exhaustion is the result of the fusion of numerous age-related impairments, and it may be the root cause of tissue and organismal ageing. Altered intercellular communication: The accumulation of damage to DNA and other cellular components can affect cellular signalling pathways. In addition, age-related chronic inflammation can also interfere with intercellular communication. Disabled macroautophagy: One of the most prominent factors for decreased cellular turnover associated with ageing is the decline in autophagy, which is the process of self-digestion. Chronic inflammation: Specific immune system and inflammation-targeting therapies can accelerate or decelerate the ageing process in certain organ systems. Microbial imbalance: Microbial dysbiosis can induce ageing through ecological imbalances in the microbiome^6^.

To detect the anti-ageing pathway of *P. vulgaris,* markers of various pathways were detected. For the oxygen-free radical hypothesis, we detected SOD2 and MDA; In response to the immune ageing hypothesis, TNF-α, splenic cell proliferation capacity and T/B/NK cell number were detected. As for the telomere pathway, we detected cyclin p16^INK4A^ and its downstream protein Rb instead. In addition, HE staining was performed on mouse liver tissues to observe the morphological changes of cells.

SOD2, also known as manganese superoxide (MnSOD), is the main antioxidant enzyme that clears ROS (especially superoxide) in the matrix of mitochondria. It serves as the first line of defence against mitochondrial oxidative damage^28^. A number of disorders will develop as a result of the body’s overproduction of superoxide. Cell apoptosis and senescence are the most significant ones^29^. According to Qiu et al.^30^, calorie restriction increases the binding of Sirtuin-3 (SIRT3) and SOD2, which is the ability of SOD2 to exert antioxidant activity. Reduced oxidative stress, ultimately increasing the mammal longevity and ameliorating various diseases. However, according to YaJie Cui et al.^31^, miR-146a enhances the production of ROS in Epithelial Ovarian Cancer (EOS) by suppressing the expression of SOD2. This increases tumour cell apoptosis, inhibits proliferation and increases sensitivity to chemotherapy. We observed that Cui et al.’s study was biased to a certain extent because it only took into account tumor cells and ignored the survival status of normal cells. In this study, the expression of SOD2 in the liver and kidney of mice was significantly increased after being treated with high-dose *P. vulgaris.* We demonstrate that this is because *P. vulgaris* may rejuvenate the mice by controlling the expression of SOD2, which helps it resist oxidative stress. It might have anti-aging properties.

Cyclin inhibitor p16^INK4A^ is one of the biomarkers of cell senescence. It is inhibited during early embryogenesis, while it is gradually induced and increasing during senescence and is a major factor causing cell senescence^32^. Baker et al.^33^ claim that drug-induced elimination of P16^INK4A^-positive senescent cells can stop or slow the onset of known age-related diseases and prolong healthy life. *P. vulgaris*, according to Shen et al.^34^, can prolong the life span of female flies by 10.42% and increase their heat stress tolerance by 18.46%. In this study, the lowest levels of p16^INK4A^ expression were found in the mouse liver following high-dose *P. vulgaris* therapy, while the lowest levels were found in the kidney following low-dose *P. vulgaris* treatment. We demonstrate that *P. vulgaris* may be organ-specific in removing p16^INK4A^-positive senile cells. Additionally, the person may develop innate immune cells and release cytokines and chemokines, leading to immune-mediated elimination of these cells^35^. Meanwhile, in the liver, we discovered that after high-dose *P. vulgaris* treatment, the nuclear membrane of hepatocytes was intact, the size of hepatocytes was uniform, the hepatic sinuses were congested, and the direction of the hepatic cord was clear. The findings presented above show that *P. vulgaris* can delay the onset of ageing in mice by removing senescent cells in a variety of ways.

Ageing is characterized by T lymphocytopenia, T cell dysfunction, and elevated serum TNF-α levels^36^. Adalimumab, an inhibiting antibody to TNF-α, can counteract the negative consequences of ageing, claim Liberale et al.^37^. According to Zhang et al.^38^, *P. vulgaris* can down-regulate TNF-α and regulate mitogen-activated protein kinase (MAPKs), transforming growth factor-β (TGF-β)/Smad signalling pathways and play a protective role in Ultraviolet B (UVB)-induced ageing normal skin fibroblasts. In addition, we measured the levels of TNF-α and MDA in serum following the *P. vulgaris* treatment and discovered that compared with the ageing group, the levels of TNF-α and MDA were lower, whereas the effect of the VE group was better. We detected immune cell proportion in whole blood cells and found that the proportion of T cells (CD3^+^/CD4^+^/CD8^+^) was the highest under the action of low-dose *P. vulgaris*, suggesting that *P. vulgaris* had a dose-dependent immune effect. However, the proportion of NK (CD3^−^, NK1.1+) cells is only raised when *P. vulgaris* is used in high doses. According to Lin et al., evidence shows that the proportion of NK cells will decline during anti-ageing, with the rise in quantity being viewed as compensatory and the harmful effect of the cells diminishing^39^. However in this experiment, we observed that the increase of NK (CD3^−^, NK1.1^+^) in this experiment is not only a rise in compensatory activity but also an increase in normal function. Consequently, *P. vulgaris* plays a role in immune anti-ageing.

Current research shows that there are two main ways to regulate cell ageing. The first is the regulation of replication senescence, which is dependent on the regulation of p53 → p21 and pRb → E2F signalling pathways, primarily because the increased level of reactive oxygen species eventually leads to DNA damage and double-stranded DNA breaks (DSB), which causes cell senescence^40^. The second is non-telomere-dependent cellular senescence induced by oxidative stress, which is regulated by the ERK → p38MAPK → p16 → pRb signalling pathway. In addition, p16 coordinates with p21 in a p53-independent ageing pathway to induce low phosphorylation of retinoblastoma protein (Rb), thereby permanently trapping cells in the G1 phase^41^, according to this pathway, the content of phosphorylated Rb was detected in this study, and it was found that after the anti-ageing effect, the level of Rb in the high-dose group was significantly decreased, which was consistent with this pathway. Therefore, we believe showed that *P. vulgaris*, still play a role in the protein of the p16^INK4A^ pathway, thus delaying the ageing of cells and showing rejuvenation. In the following research, we will aim to study the specific action pathway of *P. vulgaris* to play the anti-ageing effect.

The purpose of this study was to evaluate the effects of *P. vulgaris* on senescence, as well as its impacts on immune cells and potential mechanisms of action. In the study on anti-ageing of *P. vulgaris*, we found that there was a significant gender difference. The anti-ageing impact on female mice was not as noticeable as it was on male mice, as seen in the Supplementary Table 1. According to Shen et al.^34^, the study also proved that there were gender differences in the anti-ageing of *P. vulgaris*. We noted that these findings were likely caused by the different hormone levels found in males and females. Hye-InKim et al. and Collins et al.^42,43^ also proved that Prunella has an anti-estrogen effect. We demonstrate that Prunella may act on estrogen, which leads to the failure of Prunella itself to play an anti-ageing role. In the following work, this experiment will further explore the anti-ageing effect of *P. vulgaris* from the difference in hormone levels. According to our study, Prunella can eliminate p16-positive cells and thus rejuvenate the cells.

According to Baker et al.^44^, the Elimination of p16^INK4A^ positive cells delays the development of tumours and alleviates age-related deterioration in organs. Accumulation of p16^INK4A^ positive cells in adulthood negatively affect longevity. Our study of the flow cytometry data revealed that, the proportion of NK cells increased in the anti-ageing samples treated by *P. vulgaris*, which may prove that immune cells are eliminating p16-positive cells. According to Lowe et al., senescent liver cells can be removed by the body’s immune cells^45–47^. Increasing the number of immune cells, like NK cells, may help slow the ageing process, according to a study by He et al.^48^.

In addition, our study has certain limitations. First of all, while our study has demonstrated the anti-ageing and immune-regulating effects of *P. vulgaris*, we do not yet have extensive clinical trial data. Furthermore, we did not further assess the functionality of these immune cells; instead, we merely counted the number of immune cells in the blood. Additionally, the results of this study do not completely explain the precise molecular processes through which *P. vulgaris* exerts its anti-ageing properties. In conclusion, the study of this experiment is significant and offers a fresh clue for anti-ageing research.

## Conclusion

This is the first study to demonstrate the anti-ageing effect of *P. vulgaris*. *P. vulgaris*’s own anti-inflammatory and immune-modulating properties may also help it prevent a number of age-related diseases.

## Supplementary Information

Supplementary Figures.

Supplementary Table 1.

## Data Availability

All data generated or analysed during this study are included in this published article [and its Supplementary Information files].
