# Supplementary figures and images for: Research on the anti-ageing mechanism of Prunella vulgaris L
Source: Sci Rep. 2023 Jul 31;13:12398. doi: 10.1038/s41598-023-39609-1 (PMC10390563; doi:10.1038/s41598-023-39609-1)

**Figure1:**

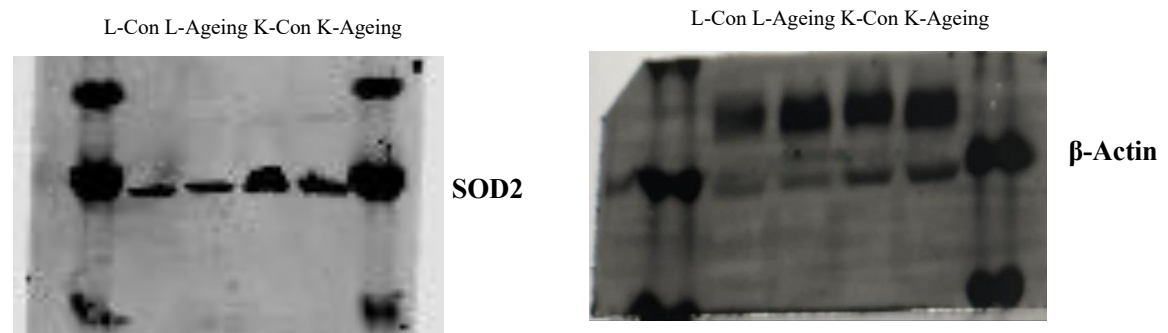

Abbreviation:

L: Liver

K: Kidney

**Figure2:**

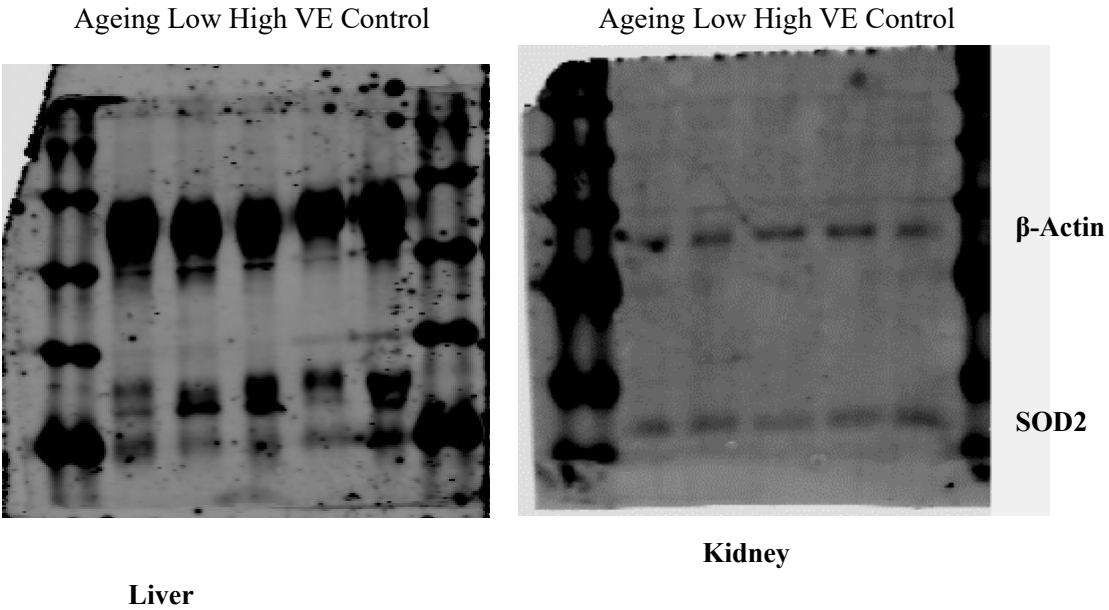

**Figure3:**

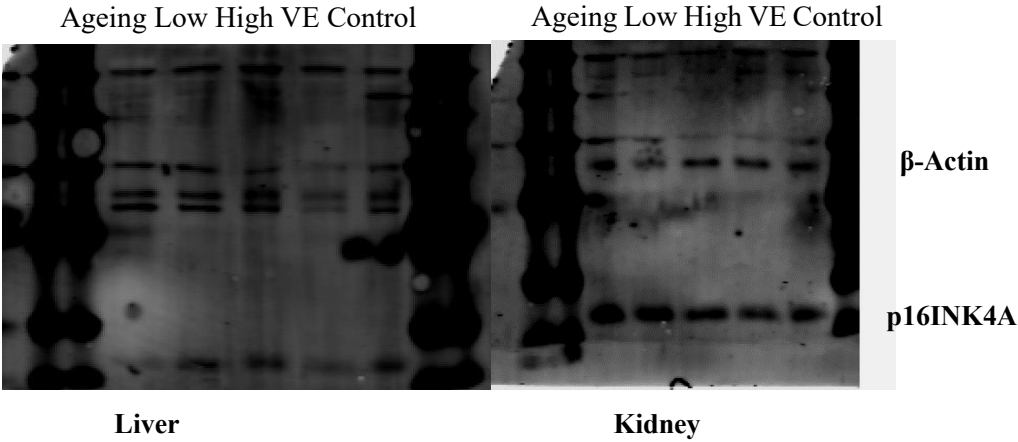

Supplement: Supplementary file 1 — Supplementary Figures. [file 41598_2023_39609_MOESM1_ESM.pdf]

**
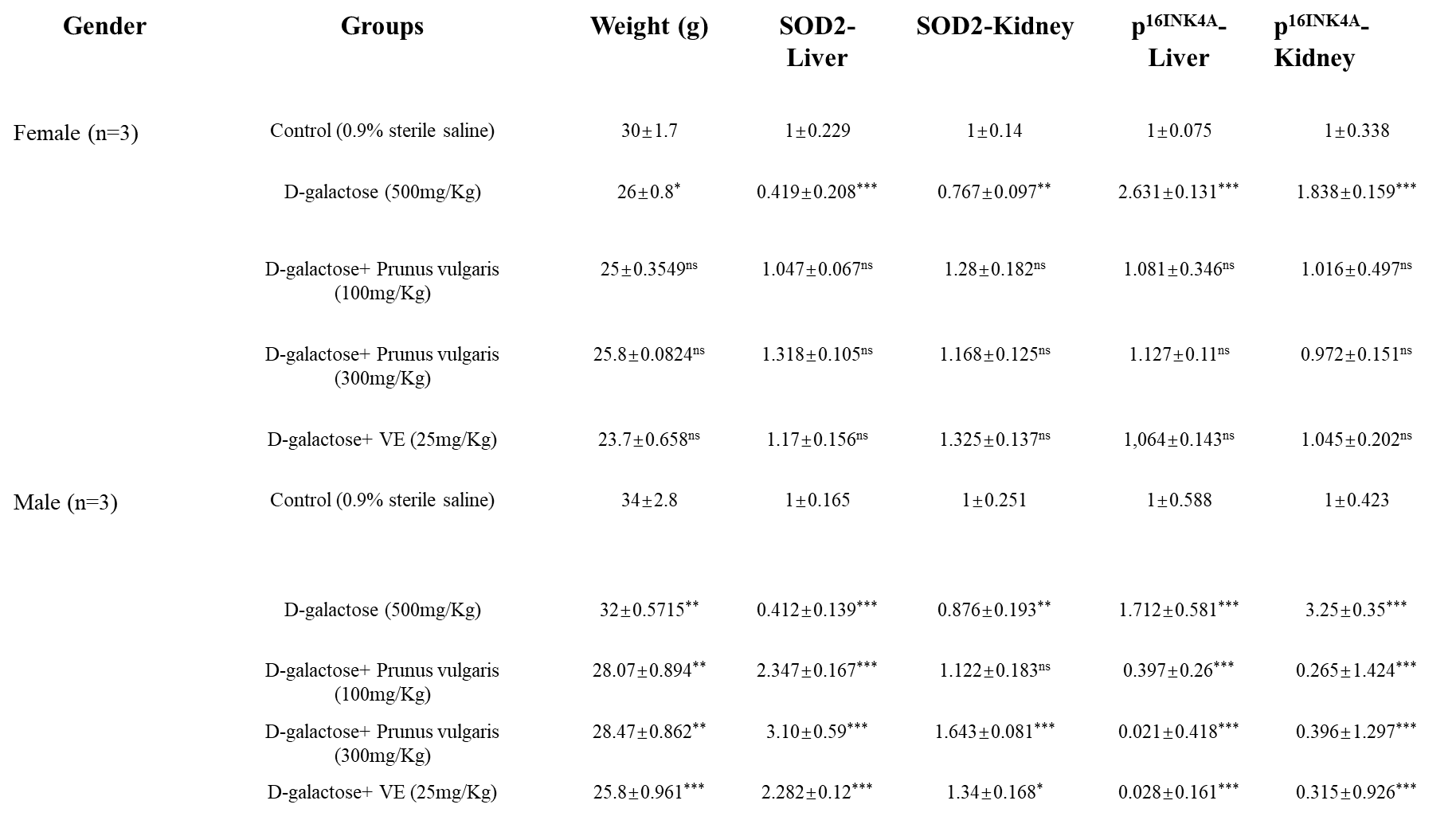
Table 1 Grouping Situation**

Where * represents (p<0.05) ** represents (p<0.01) *** represents (p<0.001).

Supplement: Supplementary file 2 — Supplementary Table 1. [file 41598_2023_39609_MOESM2_ESM.docx]
